# Serum levels of zinc, copper, selenium and glutathione peroxidase in the different groups of colorectal cancer patients

**DOI:** 10.22088/cjim.11.4.384

**Published:** 2020

**Authors:** Raghad F. Al-ansari, Abdulnasser M. AL-Gebori, Ghassan M. Sulaiman

**Affiliations:** 1 Applied Chemistry Division, Applied Science Department, University of Technology, Baghdad, Iraq; 2 Biotechnology Division, Applied Science Department, University of Technology, Baghdad, Iraq

**Keywords:** Colorectal cancer, Zn, Cu, Se, GPx, Serum

## Abstract

**Background:**

Colorectal cancer (CRC) is considered the fourth type of cancer that causes death worldwide. Changes in the levels of zinc (Zn), copper (Cu), and selenium (Se) as well as low glutathione peroxidase (GPx) activity can lead to CRC and this study was aimed to evaluate their possible use as diagnostic markers.

**Methods:**

CRC patients (n=90) were divided into three groups; newly diagnosed, before surgery, and after surgery. These groups were compared to healthy subjects (n=30); the mean age ±SD was 50.63±9.26 and 49.97±10.85 for CRC patients and healthy subjects, respectively. Biochemical study for serum levels of Zn and Cu was measured by FAAS, Se was measured by HGAAS, and ELISA for GPx.

**Results:**

Zn, Cu, Se and GPx were significantly lower in all CRC patient groups, except for the after surgery group which showed no differences for Zn and GPx as compared to the healthy subjects. Positive correlations were found between Se and Zn and between Se and GPx (r=0.71, r=0.42; P<0.01, respectively) in all CRC patient groups. A receiver operating characteristic (ROC) curve analysis was applied for the newly diagnostic group showing all the parameters that can be used as diagnostic markers for CRC.

**Conclusion:**

The present results conclude that Zn, Cu Se, and GPx can be used as diagnostic markers for CRC, where the decrease of these parameters may be associated with an increased risk of CRC and as indicators of the response to therapy.

Colorectal cancer (CRC) is considered as the fourth cause of death among types of cancer globally. It is also ranked third among the most commonly diagnosed types of cancer (1). CRC refers to malignant epithelial neoplasms that occur in the colon and/or the rectum by transforming epithelial cells into adenocarcinoma cells (2). Zinc (Zn), copper (Cu), and selenium (Se) are essential dietary nutrients for the body and are implicated in cancer risk, where they act as anti-oxidant agents. Zn catalyzes the activity of more than 300 enzymes and has roles in the immune function, DNA synthesis, protein synthesis, and cell division. It is also responsible for the maintenance of the structure of DNA and its binding to more than 1000 transcription factors that are required for gene expression of many proteins. Cu plays an important function in preserving the integrity of DNA by preventing oxidative DNA damage. Many enzymes and proteins in humans depend on Zn, Cu and Se. Both Zn and Cu play the main role in the activity of the antioxidant enzyme known as copper-zinc superoxide dismutase. Se also contributes to the formation of some enzymes such as glutathione peroxidase (GPx), thioredoxin reductase (TrxR) and iodothyronine deiodinases (IDD) which act as anti-oxidant enzymes. 

It has also an important role in the protection against oxidative stress through the action of antioxidant selenoproteins against reactive oxygen species (ROS) and reactive nitrogen species (NOS). Together, H_2_O_2_, O^2−^, and OH radicals form the ROS, the excessive generation of which causes oxidative stress. Many diseases, such as cancer, can develop as a result of oxidative stress, if there is an imbalance between the defense antioxidant system of the cell and the generation of ROS species (3-8).

A previous study on CRC patients showed that the serum level of Se decreased while Zn level increased, while it showed no difference in the level of Cu (9). Zn deficiency appeared in colon cancer patients, whereas Cu showed no difference compared with the healthy controls in a Saudi population (10). Iraqi patients with colon cancer also showed a decrease in Zn and an increase in Cu levels (11). The same findings were also reported in CRC patients in Brazil (12). Another recent study has reported that both Zn and Cu decreased in Iranian CRC patients (13).

GPx (EC 1.11.1.9) is an enzyme that is classified as oxidoreductase which catalyzes the reduction of the organic hydroperoxides or H_2_O_2_ to corresponding alcohols or water using reduced glutathione. Some GPx isozymes are described as selenium-dependent (14). Previous studies reported that GPx activity in colon cancer patients was lower in the plasma and serum as compared to the control (15, 16).

Several factors are involved in the process of CRC treatment, including the disease stage; about 95% of stage I and 65-80% of stage II patients can only be treated via surgery. However, several types of treatment such as chemo- and radiotherapy can be applied to patients in stages III and IV before having to undergo surgery (17). The ability of cancer cells to spread to other tissues, including lymph nodes, was well documented, while the rate of this process as well as the speed of cancer cells growth are correlated with the disease grades that are classified into G1, G2, and G3 according to severity (18).

Hence, the previous investigations conducted on the relationships between Zn and Cu with CRC are controversial as to whether these elements increased or decreased in the serum of the patients. Also, the cutoff values of these parameters were not defined by previous studies. In this study, we assessed the levels of these parameters in different groups of CRC patients and determined the cutoff values that could be applied for newly diagnostic patients. Also, we analyzed the correlations among the investigated elements.

## Methods

**Study population:** This research involved 30 healthy subjects (males and females) and 90 patients who were diagnosed as primary colorectal adenocarcinoma patients. The mean age was 49.97 ±10.85 years for healthy subjects and 50.63±9.26 years for CRC patients. Healthy subjects matched the patients in the gender ratio. Tumor lymph node metastasis (TNM) system was used for staging. CRC patients were divided into three equal groups; newly diagnosed (no treatment, no surgery, all stages of disease), before surgery (chemo- and radiotherapy-treated, stages III and IV), and after surgery (No treatment before and after surgery, stages I and II). Patients with diabetes, heart diseases, kidney failure, familial history for CRC, intestinal polyposis, chronic digestive problems, and those who are alcoholic and smokers were excluded. The diagnosis for CRC patient was performed by consultant doctors who identified tumor location, whereas tumor type, grade, and stage were identified by pathologists. The ethics committee of the Medical City in Baghdad, Iraq approved this research. The number of CRC patients within stage I was 23 (25.55%), stage II was 23 (25.55%), stage III was 22 (24.44%), and stage IV was 22 (24.44%). The number of patients with grade 1 was 22 (22.22%), grade 2 was 58 (64.44 %), and grade 3 was 12 (13.33%). Tumor location was determined using the International Classification of Diseases (ICD- version 10). Patients with colon and those with rectal cancers were both included, where the number of patients who had a primary tumor in the cecum was 4 (4.44%), in the ascending colon,11 (12.22%), in the hepatic flexure,5 (5.55%), in the transverse colon, 8 (8.88%), in the splenic flexure, 6 (6.66%), in the descending colon, 15 (16.66%), in the sigmoid colon, 12 (13.33%), in the recto-sigmoid junction, 10 (11.11%), and in the rectum was 19 (21.11%).

**Materials:** Chemicals used for preparing standard solutions of Zn, Cu and Se element were purchased from Merck KGaA, Germany. Enzyme linked immunosorbent assay research kit (type sandwich ELISA) was used to assay glutathione peroxidase activity purchased from MyBioSource-U.S.A.

**Laboratory assessment:** Specimens were taken from healthy subjects and CRC patients; Blood (10 mL) was collected from each person, then the serum was stored at -40º C after being separated by centrifugation. Atomic absorption spectroscopy (AAS; novAA 300, Analytik Jena, Germany) was used to assay Zn, Cu and Se, whereas flame atomic absorption spectroscopy (FAAS) was used for Zn and Cu assay, using acetylene–air as a flame and hollow cathode lamps as a radiation source. Burner height and gas flow rates were adjusted to achieve the highest absorbance signal of each element. Slit width used to isolate wavelength was 1 nm. Absorbance was read at 213.9 nm and 324.7 nm, respectively, for Zn and Cu. Hydride generation atomic absorption spectroscopy (HGAAS) was used for Se assay. Hydride generation system was heated to 950º C. Absorbance was read at 196.0 nm. Carrier gas was argon. For hydride generation, NaBH4 0.6% solution (98% Sigma, Germany) in 0.6% NaOH (HiMedia Laboratories Pvt. Ltd., India) was prepared. Samples were prepared by adding 3 mL of HNO3 70% (HiMedia Laboratories Pvt. Ltd., India) to 1 mL of serum, followed by decomposition by microwave according to a specific program for decomposition. Samples were then left for 10 min before adding deionized water to a defined volume. These steps were applied to all samples. 5 mL of HCl 37% (Sigma, Germany) was also added to samples prepared for the Se assay which were heated at 85º C for 30 min (9).

**Statistical** **analysis:** IBM SPSS statistics Version 24 was used to analyze the data by Windows 10. The parameters were expressed as mean±SD, for normal distribution Shapiro- Wilk test was used and for homogeneity of variance, Levene test was used, One-way ANOVA test was used to find the differences in the means between the groups also t-test. The cutoff values and diagnostic markers were estimated by receiving operating characteristic (ROC) curve. The Pearson correlation coefficient was used to find the correlations among the parameters. Differences at p<0.05 were considered to be significant.

## Results

**Figure 1:**
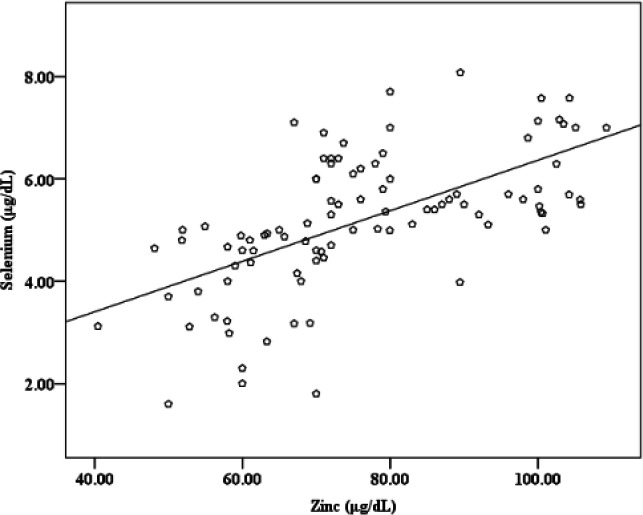
Positive correlation between Se and Zn in CRC patients

**Figure 2:**
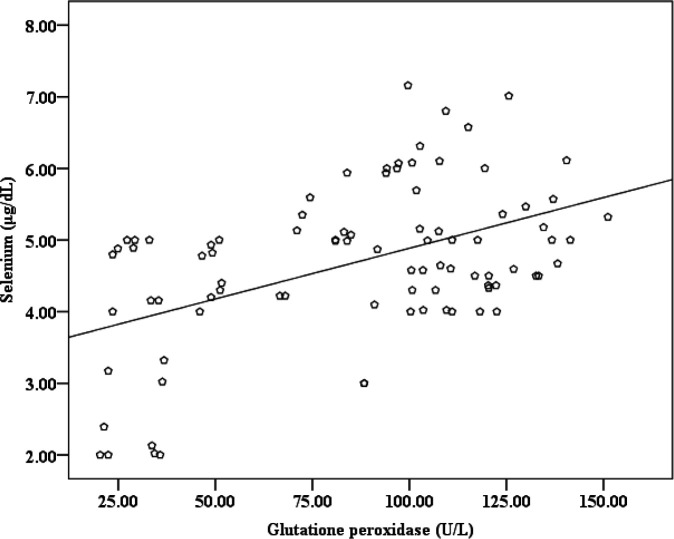
Positive correlation between Se and GPx in CRC patients

The values of all the tested parameters (Zn, Cu, Se and GPx) were significantly lower in all CRC patient groups as compared to healthy subjects, except for the after surgery group which showed no significant difference for Zn and GPx table 1. Age (years) showed no differences between CRC patient groups, newly diagnosed, before and after surgery, (50.75±11.93, 52.00±12.79, and 49.16±12.08; respectively) compared with healthy subjects (49.97±10.85). Body mass index (Kg/m^2^) also showed no differences between CRC patient groups (23.94±3.50, 24.18±4.55, and 24.02±4.03; respectively) compared with healthy subjects (24.12±2.34). The correlations among the parameters are shown in table 2, while the positive correlations between Se and Zn and between Se and GPx are shown in figures 1 and 2.

ROC curve analysis was applied for the newly diagnostic group and showed that Zn, Cu, Se, and GPx can be used as diagnostic markers for CRC disease; the area under the curve (AUC) explains the ability of using these parameters as markers. The analysis showed that all the differences in the AUC values were significant (p<0.01). For each parameter, 95%-confidence interval (95%-CI) and standard error (SE) for the AUC were calculated. The cutoff values were assessed at the maximum of both sensitivity and specificity, as shown in table 3.

Correlations between both BMI and age with the parameters in healthy subjects and CRC patients are shown in table 4. The associations of clinical features for CRC patients and the parameters are shown in table 5.

**Table 1:** Total mean serum levels of Zn, Cu, and Se, with GPx activity in the sera of healthy subjects and CRC patients

Groups	Zn (μg/dL) Mean±SD	Cu (μg/dL) Mean±SD	Se (μg/dL) Mean±SD	GPx (U/L) Mean±SD
**Healthy subjects **	98.97±4.78	80.11±3.21	10.81±1.02	136.03±4.23
**Newly diagnosed**	69.37±3.72**	30.38±2.57**	2.87±0.56**	69.73±4.94**
**Before surgery**	78.43±5.01**	47.55±3.35**	4.77±1.06**	98.35±3.63**
**After surgery **	101.23±5.32	55.50±2.48*	7.44±1.21*	133.78±5.10

*P<0.05, **P<0.01

**Table 2:** Correlations between parameters in serum for CRC patients

Correlation between	r (*P-*value)
**Zn and Cu**	-0.16 (>0.05)
**Zn and Se**	0.71 (<0.01)
**Zn and GPx**	0.24 (>0.05)
**Cu and Se**	0.01 (>0.05)
**Cu and GPx**	-0.07 (>0.05)
**Se and GPx**	0.42 (<0.01)

**Table 3:** ROC curve analysis findings for parameters

Parameters	AUC	SE	Sensitivity (%)	Specificity (%)	95% - CI	Cutoff value
**Zn**	0.91 *	0.04	89	69	0.82- 1.00	84.45 (μg/dL)
**Cu**	1.00 *	0.00	100	0	1.00- 1.00	55.24 (μg/dL)
**Se**	1.00 *	0.00	100	0	1.00- 1.00	5.40 (μg/dL)
**GPx**	0.91 *	0.05	78	84	0.80- 1.00	113.37 (U/L)

* P<0.01

**Table 4:** Correlations between both BMI and age with parameters in serum for healthy subjects and CRC patients

**Correlation between**	**CRC Patients** **r (p value)**	**Healthy subjects** **r (** ***P-*** **value)**
Zn and Age	0.03 (0.75)	-0.14 (0.53)
Cu and Age	-0.09 (0.43)	0.08 (0.73)
Se and Age	-0.005 (0.96)	0.18 (0.44)
GPx and Age	0.02 (0.87)	-0.29 (0.20)
Zn and BMI	-0.01 (0.93)	-0.22 (0.33)
Cu and BMI	-0.08 (0.50)	-0.03 (0.10)
Se and BMI	0.25 (0.04)	0.27 (0.26)
GPx and BMI	-0.01 (0.90)	0.04 (0.86)

**Table 5:** Association of clinical features for all CRC patients and serum levels of Zn, Cu, Se, and GPx

Group	Case (%)	Zn (μg/dL) Mean±SD	Cu (μg/dL) Mean±SD	Se (μg/dL) Mean±SD	GPx (U/L) Mean±SD	Sig.
**Gender** **Female ** **Male**	48.88 51.11	82.77±2.32 83.23±1.34	45.91±1.86 43.02±3.69	4.99±0.4 5.03±0.5	98.98±3.56 102.22±4.43	N.S
**Age** **≤50 ** **>50**	52.22 47.77	81.87±5.04 84.13±4.43	45.70±3.76 43.22±2.34	5.03±0.6 4.99±0.3	99.37±3.56 101.95±2.43	N.S
Therapy* **Without ** **With **	50.00 50.00	69.37±3.72 78.43±5.01	30.38±2.57 47.55±3.35	2.87±0.56 4.77±1.06	69.73±4.94 98.35±3.63	<0.001

*Only between two groups of CRC patients (the newly diagnosed and the before surgery). N.S: Non-significant

## Discussion

Alterations of trace element levels adversely affect many biological processes and they could also promote carcinogenesis. The results of our study showed that Zn, Cu, Se and GPx were significantly lower in all groups of CRC patients, while the patients in the after surgery group showed no significant difference for both Zn and GPx, as shown in table 1. 

In the present study, all groups of CRC patients were deficient in Cu and Se, as it was observed, for example, in the newly diagnosed group in all stages as well as in the patients before surgery who received radiotherapy and chemotherapy in advanced stages. In the after surgery group, the early surgical intervention in the patients with eral stage disease could not restore normal levels of the studied elements, even after 21 days of post-surgical blood collection. This indicates that tumor removal was not efficient in bringing these parameters to normal levels. A recent study on males and female patients with thyroid cancer has demonstrated that serum levels of Se significantly decreased in the pre- and post-operative patients, an effect that was suggested to be associated with thyroid cancer pathogenesis (19).

The rise in the levels of free radicals was related to cancer etiology because such a rise can damage DNA, cause destruction of proteins, and ultimately lead to tumor growth. Copper-restricted diet in humans leads to elevated fecal free radicals, and causes cytotoxicity which is one of the putative colon cancer’s risk factors (20-23).

Moreover, experiments in animals indicated that low Cu intake is considered as a risk factor for 3,2’-dimethyl-4-aminobiphenyl (DMABP)-induced colon tumor development in rats, whereas the activities of ceruloplasmin and Cu,Zn-SOD enzymes were reduced in rats fed on low Cu intake (24). Se is engaged in a number of biochemical pathways where it can be found in many forms. Anticarcinogenic pathways of Se include the prevention of oxidative damage, regulation of immune responses, repair of DNA damage, and regulation of apoptosis and cell cycle (25, 26). Selenomethionine is a major component of Se diet that modulates the redox status (reduction/oxidation) (20, 27). Besides that, it induces the P53-mediated cell cycle arrest and programmed cell death in human colon cancer cells (28). Se significantly induces apoptosis and its relatively high doses were related to overexpression of p53 in rat hepatocytes (29). A previous study reported that low serum levels of Se were strongly correlated with CRC risk (30). Zn level and GPx activity significantly decreased in the newly diagnosed and before surgery groups, but the levels showed non-significant differences in the after surgery group as compared with healthy subjects. This may be attributed to the stage and differentiation grade of the disease. Our study with the after surgery group involved patients in G1 and G2 grades only, that showed non-significant difference in these parameters as compared to the control group. 

In a study conducted on colon and rectal cancer patients of all stages of the disease who did not undergo surgical intervention or treatment, the authors reported that the levels of serum Zn significantly decreased but only in advanced stages (31).

Another study in patients with colon and stomach cancer demonstrated that high grade differentiated (G3; poorly differentiated) stomach tissue has lower Zn level comparing with the normal tissue and with the tissues from moderately differentiated carcinoma G2 and well-differentiated carcinoma G1 grades. Also, the study reported the inverse association between Zn levels in the tissues and the advanced stages of carcinoma in both colon and stomach cancer patients (32). In our study, ROC curve analysis for the newly diagnosed group was used to illustrate the association of these parameters with CRC. The findings in table 3 indicate that the parameters can be used as diagnostic markers, where a very highly significant difference in AUC is shown. These values can be used to predict people's health when the levels of these parameters are less than the cutoff values, then the individuals are at risk or already having CRC. A previous study noted that the progression to colon cancer was associated with low levels of Zn and decreased Cu,Zn-SOD activity in the plasma of rats (33). P53 folding and misfolding is modulated by Zn, which is one of the reasons that causes cancer (34). A previous study about colorectal cancer showed that the serum levels of both Zn and Cu were significantly lower as compared to healthy people (13). Human CRC patients had lower serum concentrations of Cu, Zn, and Se according to a review published in 2019 (35). Our results are consistent with these studies. Cu and Zn deficiencies have recently increased in different regions of the world for unknown reasons. The total prevalence of Cu deficiency in populations in Iran and Spain was 32.1 % (age 15-65 years old) and 30.1 % (age over 60 years old), respectively. The Spanish study also reported that the total prevalence of Zn deficiency was 66.8% (36, 37). The positive correlation between Zn and Se that we found can be explained by the results of a previous report which found that Zn can induce a decrease in Se urinary excretion (38). In a previous study on humans, two significant positive correlations between Zn and Se were found in two biological media (urine and feces) from healthy people. The study also found a significant positive correlation between dietary Zn intake and Se levels in blood (39). Hence, Zn deficiency may contribute to Se deficiency. Hypothetically, Zn may influence the status of Se by modulating one of the phases of Se homeostasis, represented by absorption, excretion or retention. We also found another correlation between Se and GPx, where Se deficiency led to decreased GPx activity, which was previously shown to result in the accumulation of H_2_O_2_, leading to destruction of the cells (14,5). In studies conducted on colon cancer patients, the GPx activity was reported to decrease in the plasma and serum (15, 16). Our results have been consistent with these recent studies.

In our study, BMI was positively associated with serum Se levels in CRC patients. A previous study found that high Se diet causes a subclinical hypothyroid response which leads to weight gain and decreases energy expenditure. But a low Se diet causes a subclinical hyperthyroid response which leads to weight loss and increases energy expenditure. Therefore, dietary Se –intake alters the energy metabolism of humans (40). Other parameters showed no association with BMI for all CRC patients groups, including patients under treatment. In a previous study on breast cancer, the patients showed a BMI that was not affected by therapy (41).

In conclusions our findings indicate the involvement of low levels of Zn, Cu, and Se as well as the low activity of GPx in the pathogenicity of CRC. Zn level and GPx activity significantly decreased in the newly diagnosed and before surgery groups, but not in the after surgery group. This may be attributed to the stage and differentiation grade of the disease. Such low levels were not observed in the control subjects, while the applied exclusion criteria could probably exclude other possible sources of such declined levels, which confirm the strict association between CRC and these parameters. Thus, we conclude that Zn, Cu Se, and GPx can be used as diagnostic markers for CRC, where the decrease of these parameters may be associated with an increased risk of CRC and as indicators of the response to therapy. 
